# Public Mental Health Crisis during COVID-19 Pandemic, China

**DOI:** 10.3201/eid2607.200407

**Published:** 2020-07

**Authors:** Lu Dong, Jennifer Bouey

**Affiliations:** RAND Corporation, Santa Monica, California, USA (L. Dong);; RAND Corporation, Arlington, Virginia, USA (J. Bouey);; Georgetown University, Washington, DC, USA (J. Bouey)

**Keywords:** COVID-19, 2019 novel coronavirus disease, SARS-CoV-2, severe acute respiratory syndrome coronavirus 2, viruses, respiratory infections, zoonoses, mental health, public health emergency, preparedness, response, China

## Abstract

The 2019 novel coronavirus disease emerged in China in late 2019–early 2020 and spread rapidly. China has been implementing emergency psychological crisis interventions to reduce the negative psychosocial impact on public mental health, but challenges exist. Public mental health interventions should be formally integrated into public health preparedness and emergency response plans.

China was the first country affected by the pandemic of 2019 novel coronavirus disease (COVID-19), caused by severe acute respiratory syndrome coronavirus 2. Several unique characteristics of China’s COVID-19 epidemic patterns and its management policy prompted a heightened public mental health crisis. First, many Chinese residents still remember the 2003 outbreak of severe acute respiratory syndrome (SARS) and its effect on China’s social life and economy (*1*). COVID-19 is more transmissible than SARS, and the case-fatality rate (2.3%) is substantially higher than that for seasonal influenza (*2*). The uncertain incubation period of the virus and its possible asymptomatic transmission cause additional fear and anxiety. Second, the government’s initial downplaying of the epidemic’s severity eroded public trust in the government’s decision-making transparency and competency. Third, unprecedented large-scale quarantine measures in all major cities, which essentially confine residents to their homes, are likely to have a negative psychosocial effect on residents (*3*). Fourth, reports of shortages of medical protective supplies, medical staff, and hospital beds in Wuhan and the surrounding areas soon followed the citywide quarantine and caused enormous concern throughout the nation. Last, a unique “infodemic”—an overabundance of (mis)information on social media (*4*) and elsewhere—poses a major risk to public mental health during this health crisis.

As during the 2003 SARS and 2014 Ebola virus disease outbreaks, generalized fear and fear-induced overreactive behavior were common among the public; both can impede infection control (*5*,*6*). In addition, psychiatric disorders, such as depression, anxiety, and posttraumatic stress disorder, developed in high-risk persons, especially survivors and frontline healthcare workers (*7*).

On the basis of these recent experiences, the National Health Commission of China released a notification on January 26, 2020, providing guiding principles of the emergency psychological crisis interventions to reduce the psychosocial effects of the COVID-19 outbreak (*8*). This notification specified that psychological crisis intervention should be part of the public health response to the COVID-19 outbreak, organized by the joint prevention and control mechanism at the city, municipal, and provincial levels, and that the interventions should be differentiated by group. The intervention workforce comprises psychological outreach teams led by psychiatrists and mental health professionals and psychological support hotline teams. An attachment to this notification further outlined the key intervention targets for 6 groups: confirmed patients, persons under investigation for COVID-19, healthcare workers, persons in immediate contact with patients, ill persons who refuse to seek care, and susceptible persons/the general public (Appendix).

The release of such policy guidance acknowledges China’s recognition of public mental health needs during the outbreak. However, the notification does not specify how different resources should be mobilized and coordinated or, more important, who should deliver which type of interventions, for which group in need, and by which delivery mode(s). The policy guidance also does not indicate operationalization of how various groups should be screened or assessed to determine the type and level of interventions to provide to each. This level of detail is needed because China lacks a well-established mental healthcare system and has no existing national-level emergency response system and designated workforce to provide the psychological crisis interventions during a national emergency or disaster (Chen X, Fu X, unpub. data, https://doi.org/10.16418/j.issn.1000-3045.20200213001) (*9*). Other major challenges to successfully implementing the emergency psychological crisis interventions include China’s severe shortage of mental healthcare providers (1.49 psychiatrists/100,000 population, and only half of these psychiatrists have attained a bachelor’s degree in medicine), unevenly distributed healthcare resources, and the limitations posed by the mass quarantine (*9*). For example, hospitals, universities, and a variety of organizations have set up numerous hotlines staffed by volunteers with varying degrees of qualification and experience (*8*). These well-meaning efforts can be uncoordinated and inadequately supervised and thus are likely to cause confusion to service consumers and inefficient use of resources.

The challenges reported in China indicate that, for many developing countries, telemedicine should be considered, given the widespread adoption of smartphones, to help remove barriers to accessing quality care for mental health. Task-shifting or -sharing (i.e., shifting service delivery of specific tasks from professionals to persons with fewer qualifications or creating a new cadre of providers with specific training) might help, especially in low-resource areas (*10*). Countries should also consider requesting support and guidance from global mental healthcare authorities and research communities through international collaborations.

Given lessons learned from past outbreaks in China and other parts of the world, public mental health interventions should be formally integrated into public health preparedness and emergency response plans to effectively curb all outbreaks. The World Health Organization’s strategic preparedness and response plan for COVID-19, however, has not yet specified any strategies to address mental health needs of any kind (*4*). As the virus spreads globally, governments must address public mental health needs by developing and implementing well-coordinated strategic plans to meet these needs during the COVID-19 pandemic.

Appendix Emergency psychological crisis interventions to address public mental health during the COVID-19 pandemic, China.

## References

[1] Bouey J. From SARS to 2019-coronavirus (nCoV): U.S.–China collaborations on pandemic response: addendum. Santa Monica (CA): RAND Corporation; 2020 [cited 2020 Mar 23]. https://www.rand.org/pubs/testimonies/CT523z2.html

[2] The Novel Coronavirus Pneumonia Emergency Response Epidemiology Team. The epidemiological characteristics of an outbreak of 2019 novel coronavirus diseases (COVID-19)—China, 2020. China CDC Weekly. 2020;2:113–22. 34594836 PMC8392929

[3] Brooks SK, Webster RK, Smith LE, Woodland L, Wessely S, Greenberg N, et al. The psychological impact of quarantine and how to reduce it: rapid review of the evidence. Lancet. 2020;395:912–20. 10.1016/S0140-6736(20)30460-8 32112714 PMC7158942

[4] World Health Organization. 2019 Novel coronavirus (2019-nCoV): strategic preparedness and response plan Feb 3, 2020 [cited 2020 Feb 7]. https://www.who.int/docs/default-source/coronaviruse/srp-04022020.pdf

[5] Shultz JM, Cooper JL, Baingana F, Oquendo MA, Espinel Z, Althouse BM, et al. The role of fear-related behaviors in the 2013–2016 West Africa Ebola virus disease outbreak. Curr Psychiatry Rep. 2016;18:104. 10.1007/s11920-016-0741-y 27739026 PMC5241909

[6] Person B, Sy F, Holton K, Govert B, Liang A, Garza B, et al.; National Center for Inectious Diseases/SARS Community Outreach Team. Fear and stigma: the epidemic within the SARS outbreak. Emerg Infect Dis. 2004;10:358–63. 10.3201/eid1002.030750 15030713 PMC3322940

[7] Mak IW, Chu CM, Pan PC, Yiu MG, Chan VL. Long-term psychiatric morbidities among SARS survivors. Gen Hosp Psychiatry. 2009;31:318–26. 10.1016/j.genhosppsych.2009.03.001 19555791 PMC7112501

[8] National Health Commission of China. Principles of the emergency psychological crisis interventions for the new coronavirus pneumonia [in Chinese] [cited 2020 Feb 7]. http://www.nhc.gov.cn/jkj/s3577/202001/6adc08b966594253b2b791be5c3b9467

[9] Liang D, Mays VM, Hwang WC. Integrated mental health services in China: challenges and planning for the future. Health Policy Plan. 2018;33:107–22. 10.1093/heapol/czx137 29040516 PMC5886187

[10] World Health Organization. Joint WHO/OGAC technical consultation on task shifting: key elements of a regulatory framework in support of in-country implementation of task shifting. Geneva: The Organization; 2007.

