# Supplementary material for: Public Mental Health Crisis during COVID-19 Pandemic, China
Source: Emerg Infect Dis. 2020 Jul;26(7):1616–8. doi: 10.3201/eid2607.200407 (PMC7323564; doi:10.3201/eid2607.200407)
Supplement: Appendix — Emergency psychological crisis interventions to address public mental health during the COVID-19 pandemic, China. [file 20-0407-Techapp-s1.pdf]

Article DOI: https://doi.org/10.3201/eid2607.200407

Public Mental Health Crisis during COVID-19 Pandemic, China

Appendix

The authors note that these documents below are not official translations provided by the Chinese government. The authors translated these documents for readers who may not read Chinese to understand the content of the notification referenced in the main text.

This technical appendix contains two parts:

1. Guiding Principles for Emergency Psychological Crisis Intervention for Outbreak of the Novel Coronavirus Outbreak

2. Attachment to the “Guiding Principles”: Key Points of Psychological Crisis Intervention for Different Groups

1. Guiding Principles for Emergency Psychological Crisis Intervention for Outbreak of Novel Coronavirus Outbreak

National Health Commission of the People's Republic of China

These guidelines should be implemented under the guidance of trained mental health professionals.

1. Organization and leadership:

The psychological crisis intervention work is led by the joint prevention and containment mechanism (leadership group, headquarters) in response to the COVID-19 epidemic at the provincial, regional, and municipal levels. This mechanism also provides the necessary coordination and financial support. The mental health professional associations and other related associations and institutions should assemble experts with experience in post-disaster psychological crisis intervention, to provide technical guidance and emergency

psychological crisis intervention under the coordination of the government's health authority.

2. Guiding principles

1) Integrate psychological crisis intervention into the overall deployment of epidemic prevention and control, with the premise of reducing potential psychological damage caused by the epidemic and promoting social stability and of adjusting the focus of psychological crisis intervention in a timely manner according to the progress of epidemic prevention and control.

2) Implement targeted interventions for different groups. Strictly protect the personal privacy of patients. Prevent secondary trauma for both providers and patients.

3. Formulate the intervention plan

1) Purpose

a. Provide mental health services for the affected population

b. Provide psychological crisis intervention for the population in need

c. Actively prevent, mitigate and contain the psychosocial impact of the epidemic

d. The management and treatment of severe mental disorders should not be interrupted

2) Tasks

a. Understand the mental health status of various groups affected by the epidemic, and timely identify high-risk groups based on the information at hand to prevent extreme events such as suicide and other impulsive behaviors. Surveillance of early signs of group psychological crisis, timely reporting to the joint mechanism (lead group, command) of epidemic containment, and provide solutions.

b. Comprehensively apply various psychological crisis intervention techniques and combine them with publicity and education to provide mental health services.

c. Provide supports including training to social organizations to supply mental health services.

d. Continue the management, treatment and community care of at-home patients with severe mental disorders.

3) Determine the target population and number.

Population affected by the COVID-19 outbreak is divided into four groups. The focus of intervention should start with the first-tier population and gradually expand. General communication and education should reach all four tiers of the population.

1. First-tier population: patients confirmed with COVID-19 (hospitalized patients with severe illness and worse), front-line medical staff, front-line disease control staff, and front-line management staff

2. Second-tier population: mild patients (close contacts, suspected patients) isolated at home, patients with fever who came to the hospital for treatment.

3. Third-tier population: people related to the first-tier and second-tier population, such as family members, colleagues, friends, epidemic responders, including field commanders, organization managers, and volunteers.

4. Forth-tier population: residents of geographic areas affected by the epidemic, susceptible population, and general public who are affected by the epidemic prevention and control measures.

4) Evaluation of target population and formulation of differentiated intervention plan. Assess the mental health of the target population, identify and distinguish between the high-risk population and the general population in a

timely manner; conduct psychological crisis interventions for the high-risk population and provide mental health education for the general population.

5) Develop work schedules. Based on the scope and size of target groups and the available number of psychological crisis intervention personnel, arrange work and develop work schedules.

4. Form a team

1. Psychological intervention medical team. Can be formed as standalone team or be part of the general medical team. The staff is mainly psychiatrists, with clinical psychologists and psychiatric nurses participating. Those with experience in psychological crisis intervention are preferred. When teaming alone, one team leader is assigned, and one liaison is assigned to take charge of the team's logistical support and contact with all parties.
2. Psychological assistance hotline team. Mainly mental health workers who have received psychological hotline training and volunteers who have experience in psychological crisis intervention in public emergencies. Before taking up the job, they should receive psychological assistance training on COVID-19 outbreak and organize experts to provide supervision to the hotline staff.

5. Working methods

1. Psychiatric and mental health experts conduct timely research and judgment based on the epidemic development and the psychological state of the population, and provide decision-making recommendations and consultation for the joint mechanism (lead group, headquarters), and provide training and supervision for personnel participating in the psychological crisis intervention, and provide mental health education for the public.
2. Give full play to the role of "Healthy China", "12320", provincial health platform, existing psychological crisis intervention hotline and multiple online communication methods, coordinate the rotation of psychological workers, and provide 24/7 online services. Provide real-time psychological support to the third- and fourth-tier populations in a timely manner, and provide

supplementary psychological assistance services to the first- and second-tier groups.

3. Extensive mobilization of the society to provide social support based on the needs and difficulties of various groups affected by the epidemic.

2. Attachment to the "Guiding Principles": Key Points of Psychological Crisis Intervention for Different Groups

1. Confirmed patients
    a. Early stage of isolation.
        i. Typical presentation: numbness, denial, anger, fear, anxiety, depression, disappointment, complaining, insomnia, aggression, etc.
        ii. Interventions:
            1. Understand that the patients' emotional responses are normal responses under stress. Be prepared in advance and not be irritated by patients' aggressive or depressive behaviors and lose the stance of professional healthcare provider, such as arguing with the patient or being overly involved.
            2. Under the premise of understanding the patient, psychological crisis intervention should be given in addition to medical treatments, such as timely assessment of suicide risk, self-injury risk, risk of aggressive behavior, positive psychological support, and avoid direct conflict with the patient. Seek a psychiatric consultation if necessary. Explain the importance and necessity of isolation and encourage patients to build confidence in recovery.
            3. Emphasize that isolation is not only a way to better observe and treat patients, but also a way to protect loved ones and the society. Explain the treatment plans, and the effectiveness of the intervention.

iii. Principles: Support and comfort. Treat patients with empathy, stabilize patients' emotions, and evaluate risks of suicide, self-injury, and aggressive behaviors early.

b. Treatment stage of isolation.

i. Typical presentation: In addition to the above-mentioned possible presentation, there may also be loneliness, or lack of cooperation due to fear of the disease, abandonment of treatment, or excessive optimism and high expectations of treatment.

ii. Interventions:

1. According to the patient's level of acceptance, objectively and truthfully explain the disease and the epidemic situation, so that the patient is informed;

2. Assist the patient with communicating with loved ones and convey information if needed;

3. Actively encourage patients to cooperate with all treatment activities;

4. Make the environment suitable for the patients undergoing treatments;

5. If necessary, please consult a psychiatric consultation.

iii. Principles: Actively communicate information and consult with psychiatrists if necessary.

c. Patients with respiratory distress, extreme anxiety, and difficulty expressing themselves.

i. Typical presentation: near-death experiences, panic, despair, etc.

ii. Intervention measures: While calming and soothing, strengthen the treatment of the primary illness and reduce symptoms.

iii. Principles: soothing, sedation, pay attention to emotional communication, and enhance confidence in treatment.

d. Mild patients isolated at home, patients with fever who came to the hospital for treatment.

i. Typical presentation: panic, restlessness, loneliness, helplessness, depression, pessimism, anger, nervousness, stress from being alienated from others, grievance, shame, or disregard for illness.

ii. Interventions:

1. Assist patients to understand the situation with reliable information and knowledge, and trust scientific and authoritative medical sources;

2. Encourage active cooperation with treatment and isolation measures, healthy diet, balancing work and rest. Encourage soothing activities such as reading, listening to music, and communication using modern communication methods as well as other daily activities;

3. Accept isolation, mindful of their own reactions, and look for silver lining in adversity;

4. Seek social support to cope with stress: use modern communication methods to contact relatives, friends, colleagues, etc., and maintain social communication, for support and encouragement;

5. Encourage the use of psychological assistance hotline or online psychological intervention resources.

iii. Principles: Health education, encourage cooperation and adaptation to change.

2. Suspected Patients

a. Typical presentation: fluke-minded, avoiding treatment, fear of being discriminated against, or anxiety, seeking excessive treatment, frequent transfers among hospitals, etc.

b. Interventions:

i. policy education, close observation, and early treatment;

ii. adopt necessary protective measures;

iii. obey the overall public health strategy and report personal circumstances in accordance with regulations;

iv. adopt self-stress relieve methods and reduce stress

c. Principles: Prompt education, adopt correct way of self-protection, obey the overall strategy, and reduce stress.

3. Health care and related personnel:

a. Typical presentation: Excessive fatigue and tension, exhaustion, anxiety, insomnia, depression, sadness, grievance, helplessness, down, frustration or self-blame in the face of patient death. Fear of being infected, concerned about family members, concerned about family members worried about themselves. Excessive excitement, refusal of reasonable rest, cannot well ensure their own health, etc.

b. Interventions:

i. Conduct psychological crisis intervention training before participating in rescue, understand stress response, and learn how to respond to stress and regulate emotions. Conduct preventive interviews and openly discuss inner feelings; support and comfort; resource mobilization; help parties prepare psychologically for stress.

ii. Eliminate the worries of frontline medical workers, arrange special personnel for logistical support, and staff in the quarantine area should rotate as much as possible every month.

iii. Reasonable scheduling, arrange appropriate relaxation and rest, and ensure adequate sleep and diet. Try to arrange frontline staff at designated hospitals to stay near the hospital.

iv. Try to maintain contact and communication with family and the outside world when possible.

v. In case of insomnia, depression, or anxiety, you can seek professional psychological crisis intervention or mental health services. You can call the psychological assistance hotline or provide online psychological services, and face-to-face psychological crisis intervention can be performed in areas where conditions permit. It does not relieve for 2 weeks and affects the workers. It needs to be evaluated by a psychiatrist.

vi. If stress symptoms have occurred, you should promptly adjust your work position and seek professional help.

c. Principles: Rotate regularly, self-regulate, and ask for help if you have questions. 24.

4. Those who are in close contact with the patient (family members, colleagues, friends, etc.)

a. Typical presentations: avoidance, restlessness, anxiety during the waiting period; or blind bravery, refusal to protect and home observation.

b. Interventions:

i. Policy and education, encourage facing the reality, cooperate with home observation;

ii. Correct information dissemination and communication, release tension.

c. Principles: Mission, comfort and encourage communication through the Internet.

5. Patients who do not want to seek medical treatment

a. Typical presentations: fear of being misdiagnosed and isolated, lack of awareness, avoidance, neglect, anxiety, etc.

b. Interventions:

i. Education about the epidemic to eliminate fear;

ii. Advocate that seeking medical treatment early will benefit others;

iii. Eliminate public shaming and promote disease prevention based on scientific evidences;

c. Principles: explain and persuade, not criticize. Support patients seeking medical treatments.

6. Susceptible people and the general public

a. Typical presentations: panic, fear to go out, excessive disinfection, disappointment, fear, irritability, aggressive behavior, ftextreme optimism or pessimism, etc.

b. Interventions:

i. Provide reliable information as well as information about further disease control methods and medical services;

ii. Ensure communication, provide guidance on how to adapt to changes;

iii. Do not discriminate against diagnosed or suspected patients;

iv. Advocate against unhealthy coping methods (such as drinking, smoking, etc.);

v. Health education so the general public can identify symptoms themselves.

c. Principles: Health education, positive reinforcement, eliminate fear, prevention based on scientific evidences.
